# The Hypoxia-Inducible Factor Pathway in Adipocytes: The Role of HIF-2 in Adipose Inflammation and Hypertrophic Cardiomyopathy

**DOI:** 10.3389/fendo.2015.00039

**Published:** 2015-03-23

**Authors:** Qun Lin, Zhong Yun

**Affiliations:** ^1^Department of Therapeutic Radiology, Yale School of Medicine, New Haven, CT, USA

**Keywords:** adipocyte, cardiomyopathy, heart, hypertrophy, hypoxia, HIF-2, obesity, von Hippel–Lindau

## Abstract

Under obese conditions, adipose tissue can become oxygen-deficient or hypoxic. Extensive work has been done using various diet-induced obesity models to demonstrate an important role of hypoxia-induced signaling in adipose tissue and its impact on adipose functions related to adipogenesis, insulin sensitivity, and inflammation. We have recently identified a new mechanism connecting activation of the hypoxia-sensing pathway manifested by hypoxia-inducible factor (HIF) 2α to adipose tissue inflammation and hypertrophic cardiomyopathy. Interestingly, this observation is consistent with the clinical evidence showing that obesity is often associated with ventricular hypertrophy and dysfunction as well as congestive heart failure independent of other well-established risk factors including diabetes, hypertension, and coronary artery disease. This brief review will discuss the currently published genetic mouse models to determine the role of the HIF pathway in adipose tissue-associated diseases with a focus on the newly identified role of adipocyte HIF-2 in the development of hypertrophic cardiomyopathy.

## Introduction

Molecular oxygen (O_2_) is essential for maintaining normal tissue functions from cellular energy production to regulation of a multitude of intracellular signal transduction pathways. Under obese conditions, O_2_ concentrations in adipose tissue can be negatively affected by a number of factors. Pathological obesity is often associated with adipocyte hypertrophy (1–3). Enlarged adipocytes can reduce the effective range of O_2_ diffusion in adipose tissue. On the other hand, O_2_ supply to adipose tissue is reduced because of decreased capillary density as well as subdued blood flow in obese subjects compared to lean subjects [see reviews by Ye (4) and Trayhurn (5)]. As shown by a recent study, obesity and high fat diet (HFD) can also increase O_2_ consumption in adipocytes likely due to uncoupled respiration induced by free fatty acids (6). As a result of these and other changes, insufficient oxygenation, i.e., hypoxia occurs in adipose tissue under pathologically obese conditions.

Using a needle-type pO_2_ microelectrode to directly measured tissue pO_2_ concentrations, Ye et al. have found that the average pO_2_ is low at 15.2 mmHg in adipose tissue of the genetically obese *ob/ob* mice compared to the average of 47.9 mmHg in that of lean mice (7). Decreased adipose pO_2_ has further been independently confirmed in HFD-induced obese mice and *ob/ob* mice (8, 9). Adipose tissue hypoxia is also found in humans. The mean adipose pO_2_ in overweight or obese patients is approximately 15% lower than that of lean subjects; adipose pO_2_ decreases even further with increasing body fat percentage (10). Such direct evidence indicates that adipose tissue hypoxia is a common pathological feature of obese subjects. In contrast, other studies have found no evidence of obesity-associated hypoxia in human abdominal subcutaneous fat (11, 12). These discrepancies in adipose tissue oxygenation are likely due to the use of different pO_2_ measurement techniques [see review by Hodson (13)] as well as fat depot-dependent differences in O_2_ supply and consumption.

Increasing amounts of evidence nonetheless suggest that hypoxia can exert profound impact on adipose tissue function. It has been shown that hypoxia inhibits adipogenic differentiation (14, 15), which may further enhance adipocyte hypertrophy due to inadequate *de novo* adipogenesis (16). Hypoxia affects glucose homeostasis, lipid metabolism, and production of adipokines and pro-inflammatory cytokines in adipose tissue (7, 10, 17). It has also been shown that macrophages are preferentially localized in hypoxic regions of adipose tissue with obesity (8). These observations strongly suggest adipose tissue hypoxia is an important etiological entity closely involved in onset and/or progression of obesity-associated diseases.

## The Hypoxia-Inducible Factor Pathway

**Figure 1:**
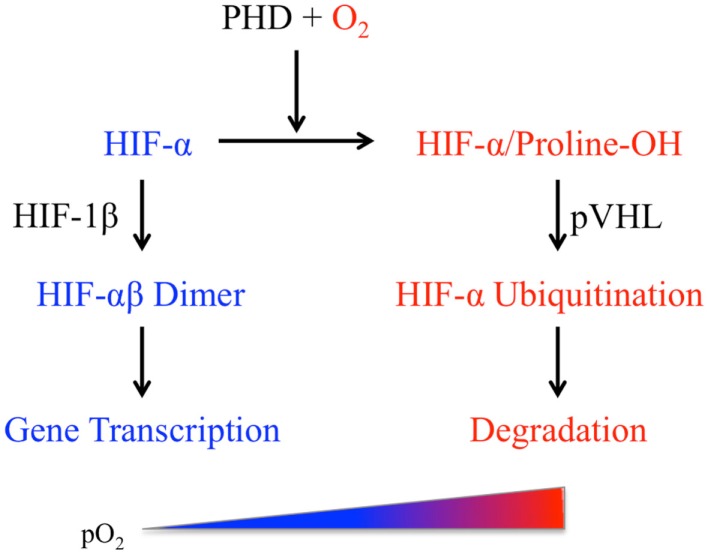
**The hypoxia-inducible factor (HIF) pathway (see text for details)**.

Mammalian cells respond to pO_2_ variations via the canonical multi-step O_2_-sensing pathway leading to the eventual activation of the hypoxia-inducible factors (HIF), a class of heterodimeric transcription factors containing the basic helix-loop-helix and PER/SIM/aryl hydrocarbon receptor nuclear translocator (ARNT) (bHLH-PAS) domains (Figure 1). Each heterodimer consists of an O_2_-sensitive HIF-α (HIF-1α or HIF-2α) and the O_2_-insensitive HIF-1β subunit (18). The quintessential aspect of this pathway is the O_2_-dependent regulation of HIF-α protein stability. Changes in pO_2_ are first “sensed” by HIF prolyl hydroxylases (PHDs), a family of O_2_-binding dioxygenases (19–22). In mammals, HIF-α proteins are regulated primarily by three PHD isoforms (PHD1, 2, and 3) among which PHD2 is the most abundant and widely expressed hydroxylase (23, 24). Under normoxic conditions, PHDs catalyze hydroxylation of the two conserved proline residues within the O_2_-dependent degradation domain (ODD) of HIF-1α or HIF-2α subunit. The hydroxylated HIF-α proteins interact with the von Hippel–Lindau tumor suppressor protein pVHL, undergo polyubiquitination, and are finally degraded by proteasomes (25). Under hypoxic conditions (e.g., <2% O_2_), PHDs are rendered inactive and HIF-α is no longer hydroxylated. The stabilized HIF-α forms a dimer with the constitutively expressed HIF-1β to activate transcription of a wide range of genes including those involved in the regulation of angiogenesis, metabolism, and inflammation (18, 26).

Murine *Hif1a* mRNA is expressed in both mature adipocytes and progenitor cells (14, 15), whereas both *Hif2a* mRNA and HIF-2α protein are found in differentiated adipocytes even under normoxic conditions (14), suggesting that HIF-2α might have a unique role in mature adipocytes. Animal model studies have shown that HIF-1α protein levels (7), as well as HIF DNA-binding activities (27), are elevated in adipose tissue of obese mice. In addition, HIF-2α protein levels are also increased in adipose tissue of mice fed with HFD for 4 weeks (28). However, activation of HIF-1 leads to suppression of adipogenesis (14, 15). Collectively, these observations suggest that the HIF pathway can play a potentially significant role in obesity, diabetes, and metabolic syndrome (29).

The efforts to study the whole-body effects of the HIF-dependent O_2_-sensing pathway have been hampered by the embryonic lethality of homozygous deletion of the key genes of this pathway including *Hif1a*, *Hif2a*, *Hif1b*, *Phd2*, and *Vhl*. Several genetic mouse models have nonetheless demonstrated a global impact of the HIF pathway on body weight and metabolism. *Hif1a*^+/−^ mice are viable but gain slightly less weight under normoxia but lost significantly more weight when maintained at 10% ambient O_2_ compared to wild-type littermates (30). Zhang et al. (31) have found that glucose increases HIF-2α protein synthesis and stability in proopiomelanocortin (POMC) neurons. Genetic deletion of *Hif1b* in POMC neurons facilitates obesity development via impaired hypothalamic glucose sensing (31). On the other hand, the PHD2 hypomorphic (*Hif-p4h-2^gt/gt^*) mice have less adipose tissue, smaller adipocytes, but improved glucose tolerance and insulin sensitivity, as well as less adipose tissue inflammation compared to their wild-type littermates (32). In this review, we will focus on the adipocyte-specific knockout mouse models.

## Genetic Mouse Models to Investigate the Role of the HIF Pathway in Adipocytes

A number of genetic approaches have been used to either inactivate or activate the HIF pathway in adipocytes. The tissue-specific gene deletion or knockout is achieved by adipocyte-targeted expression of the cre recombinase under the transcriptional control by the gene promoter of fatty acid binding protein 4 (Fabp4) or aP2. The majority of currently published work uses either of the two aP2-cre mouse strains: one is developed by Dr. Ronald M. Evans’ group of the Salk Institute (33) and commercially available from the Jackson Laboratory (#005069), and the other is created by Dr. Barbara B. Kahn’s group of the Beth Israel Deaconess Medical Center and Harvard Medical School (34). Although the same 5.4-kbp aP2 gene promoter/enhancer region was used to generate the aP2-cre construct, the two strains of aP2-cre transgenic mice appear to show differences in tissue-specific expression of cre, which is likely due to different sites of integration of the aP2-cre transgene in the moue genome. Two reports have shown that the Evans/aP2-cre strain has leaky expression in brain and liver during embryonic development (35, 36). As an alternative to the *aP2* promoter, the promoter of adiponectin (*Apn*), another adipocyte-specific gene, has been used to generate transgenic mice with adipocyte-targeted gene expression (9). Although *Apn* expression appears to be more restricted to adipocytes than *aP2*, both genes can be expressed albeit at lower levels in other tissues. Transcription of *Apn* and *aP2* also appears to be differentially regulated in adipocytes (37–39). Therefore, it is highly possible that the phenotypes of mice derived from the *Apn*-transgenic mice would not be identical to those derived from the *aP2*-transgenic mice. It is also worth noting that the promoter/enhancer fragment used for controlling transgene expression contains limited numbers of enhancer elements only and thus is not likely to fully recapitulate the expression profile of the corresponding endogenous gene.

## HIF Inactivation in Adipocytes

The direct role of the HIF pathway in adipocytes in live animals has been examined using two transgenic mouse approaches: adipocyte-targeted expression of a dominant-negative HIF (dnHIF) and cre-mediated deletion of individual HIF genes in adipocytes. Two groups generated mouse models with targeted deletion of HIF-1β, a common binding partner for both HIF-1α and HIF-2α, in adipocytes by crossing mice carrying floxed *Hif1b* alleles (Hif1b^f/f^) with the Kahn/aP2-cre mice (40, 41). Both groups found that mice with adipocyte-targeted *Hif1b* deletion were lean with reduced fat formation and were protected from HFD-induced glucose intolerance compared to wild-type littermates under the same conditions (40, 41). Adipose expression of glucose transporter 1 (Glut1) was decreased (40, 41) and glucose uptake by adipocytes was also reduced (41) in the adipocyte-targeted *Hif1b* knockout mice. Furthermore, both expression of vascular endothelial growth factor (*Vegf*) and vascular permeability were decreased in fat tissues of the adipocyte-targeted *Hif1b* knockout mice although vascular density was unchanged in fat (41). These independent results demonstrate an important role of HIF-1β in the regulation of formation, development, and function of adipocytes. However, it should bear in mind that HIF-1β, also known as ARNT, can also interact with a number of other proteins (42, 43) and therefore, some of the HIF-1β-associated phenotypes may be unrelated to the HIF pathway.

As discussed above, functions of the HIF pathway are manifested primarily by HIF-1 and/or HIF-2. A recently published work (6) examined the respective and collective roles of HIF-1α and HIF-2α in adipocytes using mouse models with adipocyte-targeted deletion of either *Hif1a* (HAKO) or *Hif2a* (H2AKO) as well as double knockout of both Hif1a and Hif2a (DHKO) that were generated by crossing with the Evans/aP2-cre transgenic mice. The main phenotypes of HAKO mice are in good agreement with an independent study using a similar HIF-1α knockout mouse model generated from the Evans/aP2-cre transgenic mice (44). The body weights of HAKO and H2AKO mice were, by and large, comparable to that of wild-type littermates fed with regular chow or HFD, which was somewhat inconsistent with the lean phenotype of the adipocyte-targeted *Hif1b* knockout mice (40, 41). Nonetheless, the vascular density in adipose tissue was little changed in HAKO mice, just like that of the *Hif1b* knockout mice (41). Interestingly, HAKO mice were refractory to HFD-induced insulin resistance, which was also found in mice with adipocyte-targeted deletion of HIF-1α generated by cross-breeding with the Kahn/aP2-cre mice (40). Furthermore, HAKO mice resisted the development of adipose inflammation induced by HFD as compared to their wild-type littermates (6). In contrast to adipocyte-targeted deletion of HIF-1α, loss of HIF-2α in adipocytes resulted in more pronounced HFD-induced inflammation, glucose intolerance, and insulin resistance compared to wild-type littermates (6). On the other hand, DHKO mice with adipocyte-targeted deletion of both *Hif1a* and *Hif2a* exhibited the same phenotype as that of the HAKO mice. These results suggest that HIF-1α and HIF-2α have opposing functions in adipocytes with HIF-2α playing a protective role whereas HIF-1α facilitating adipose tissue pathology under HFD conditions.

Alternatively, Zhang et al. (45) created a transgenic C57Bl/6xCBA mouse model in which a dominant-negative HIF-1α (dnHIF-1α) protein lacking amino acids 30–389 was selectively expressed in adipocytes under the transcriptional control by the 5.4-kpb *aP2* promoter/enhancer region. The dnHIF-1α transgenic mice gained weight more quickly and had more body fat mass than the wild-type littermates did on either regular chow or HFD (45). These mice also developed glucose intolerance and insulin resistance especially when fed with HFD. Furthermore, the dnHIF-1α transgenic mice exhibited increased sizes of lipid droplets but decreased mitochondrial biogenesis and angiogenesis in interscapular brown adipose tissue (BAT) (45). These findings are somewhat opposite to those from the *Hif1a* knockout models (6, 40, 41). It is possible that dnHIF-1α retains some of the HIF-1α functions and thus cannot fully recapitulate the genetic loss of *Hif1a*.

Sun et al. generated a double transgenic mouse (C57BL/6) model with doxycycline (DOX)-inducible overexpression of the dnHIF-1α protein lacking amino acids 30–389 (TRE-dnHIF-1α) under the control by the adiponectin promoter-driven-rtTA transgene (Apn-rtTA) (9). In contrast to the *aP2* promoter-driven dnHIF-1α (45), *Apn* promoter-driven overexpression of dnHIF-1α in adipose tissue results in decreased body weight gain and adipocyte size but increased energy expenditure and improved insulin sensitivity in the transgenic mice on HFD (9). These inconsistencies between these two transgenic studies are likely due to differences in promoters driving the same dnHIF-1α and modes of gene regulation, i.e., constitutive versus DOX-induced expression. Nonetheless, the observations made by Sun et al. are consistent with those found in mice with adipocyte-targeted *Hif1b* deletion (40, 41). Also in agreement with the *Hif1a* knockout study (6), DOX-induced overexpression of dn-HIF-1α reduced local adipose tissue inflammation in HFD-fed transgenic mice (9).

## HIF Activation in Adipocytes

Halberg et al. generated a transgenic FVB mouse model with adipocyte-targeted expression of a constitutively active HIF-1α mutant lacking amino acids 401–603 (ODD) under the control of the 5.4-kbp *aP2* promoter/enhancer (27). Expression of the HIF-1α-ΔODD mutant varied significantly in a depot-dependent manner with, relatively speaking, the highest level of expression in subcutaneous fat and the lowest in epididymal fat (27). Consistent with high transgene expression, there was a significant increase in the sizes of subcutaneous adipocytes. The hemizygous transgenic mice gained more weight than their wild-type littermates did on either regular chow diet or HFD. Plasma glucose concentrations of the HIF-1α-ΔODD transgenic mice were moderately higher than those of their wild-type littermates during the oral glucose tolerance test. Furthermore, overexpression of HIF-1α-ΔODD in adipocytes resulted in local inflammation and fibrosis in adipose tissue (27). It is quite surprising that some of the phenotypes exhibited by transgenic mice expressing the constitutively active HIF-1α lacking amino acids 401–603 are rather similar to those displayed by transgenic mice expressing the dnHIF-1α without amino acids 30–389 (9, 45). It remains to be seen whether the unexpectedly similar results from these two opposite approaches could be due to the shared C-terminal domain of HIF-1α in addition to other genetic differences in these two transgenic strains.

In contrast to the transgenic approach, the endogenous HIF pathway can be activated as a result of genetic deletion of a HIF prolyl 4-hydroxylase or the VHL gene, which leads to stabilization of both HIF-1α and HIF-2α proteins. Being the most commonly expressed member of the PHD family, *Phd2* has been targeted for genetic deletion. Matsuura et al. generated a mouse model (*Phd2^f/f^/ap2-Cre*) with adipocyte-targeted deletion of *Phd2* using the Evans/aP2-cre transgenic mice (46). The epididymal white fat pads of the *Phd2^f/f^/ap2-Cre* mice weighed less and the adipocytes were smaller in size compared to wild-type littermates. The *Phd2^f/f^/ap2-Cre* mice were resistant to HFD-induced obesity and exhibited improved glucose homeostasis. Expression of pro-inflammatory cytokines in epididymal white fat was comparable between *Phd2^f/f^/ap2-Cre* mice and their wild-type littermates under the same HFD conditions, although macrophage infiltration appeared to be reduced in epididymal white fat *Phd2^f/f^/ap2-Cre* mice (46).

Using the same genetic approach, Michailidou et al. have made quite different observations (47). Mice with deletion of *Phd2* in adipocytes (aP2-*Phd2*KO) gained more weight mainly with larger white adipose tissue mass and bigger adipocytes than their wild-type littermates fed with the same chow diet. The increased adiposity correlated with enhanced angiogenesis in the aP2-*Phd2*KO adipose tissue. Nonetheless, the aP2-*Phd2*KO mice showed no significant differences in glucose homeostasis but basal lipolysis was significantly lower compared to that of wild-type controls (47).

In addition to PHD2, PHD1 and PHD3 can also catalyze proline hydroxylation of HIF-1α and HIF-2α proteins. Therefore, possibilities exist that loss of PHD2 might, at least, be partially compensated by PHD1 and/or PHD3 in adipocytes. As reported by Matsuura et al., *Phd3* mRNA levels increased by several fold whereas the increase of *Phd1* mRNA levels was not significant in white fat of *Phd2^f/f^/aP2-Cre* mice (46). Furthermore, expression of *Hif1a* and *Hif2a* mRNA was increased more than twofold in adipose tissue of the *Phd2^f/f^/aP2-Cre* mice (46). In contrast, Michailidou et al. found that expression of *Phd1* and *Phd3* mRNA in adipose tissue was not affected in their aP2-*Phd2*KO mice (47). Using the murine 3T3-L1-derived adipocytes, Floyd et al. found that pharmacological inhibition of PHD activity did not consistently affect HIF-1α protein levels, suggesting that other pathways might also be involved in HIF activation (48). These differences, among others, may partially explain why there are so many discrepancies between these two studies despite their nearly identical genetic approach using the same Evans/aP2-cre transgenic mice from the Jackson Laboratory and the same congenic C57Bl6 genetic background.

In contrast to the multiple isoforms of PHDs, there is only one VHL gene in mammals. Therefore, genetic deletion of the VHL gene would be a more efficient approach to activate the entire HIF pathway without compensation by other isoforms. We created a genetic mouse model (fatVHLko) with adipocyte-targeted deletion of *Vhl* by crossing the *Vhl^f/f^* mice with the Kahn/aP2-cre transgenic mice (17). Consistent with reports from other groups who used the Kahn/aP2-cre transgenic mice (34, 36, 49, 50), genetic deletion of *Vhl* was highly restricted to adipocytes. Both brown and white adipocytes of the homozygous fatVHLko mice were enlarged in size, consistent with the findings from the aP2-*Phd2*KO mice (47). The fatVHLko mice showed normal fetal development and no apparent defects at birth. Interscapular brown fat developed normally although intracellular fat contents were significantly increased. In contrast, visceral white fat was generally reduced, suggesting a depot-dependent effect of HIF activation or *Vhl* deletion on adipose development. It is worth noting that reduced white fat development of fatVHLko mice (17) is similar to that found in *Phd2^f/f^/ap2-Cre* mice (46). Because hypoxia or HIF activation can suppress adipogenic differentiation of preadipocytes (14, 15), it is possible that white fat tissue development may be more sensitive than brown fat tissue to HIF-dependent inhibition of adipogenesis. This concept is further supported by the finding that levels of *Hif1a* and *Hif2a* mRNA were significantly increased in white fat tissue of *Phd2^f/f^/aP2-Cre* mice (46), suggesting that further elevated HIF activation in adipocytes of *Phd2^f/f^/ap2-Cre* mice potentially contributes to reduction of white adipose tissue.

## HIF-2, Adipose Inflammation, and Hypertrophic Cardiomyopathy

Some 50 years ago, it was reported (51) that increase in heart weight above the predicted normal value was proportional to the increase of body weight over the normal range and grossly obese patients presented with ventricular hypertrophy as the predominant and most specific alteration in the heart. Ensuing studies further found that obese patients often developed cardiomyopathy with increased wall thickness and cavity volume of the left ventricle (52). As discussed below, activation of the hypoxia-sensing pathway in adipocytes could potentially be a leading cause of obesity-associated cardiomyopathy.

As our study has shown, all fatVHLko mice developed cardiomegaly within 7 days after birth, while all other major organs appeared to develop normally (17). Incidentally, this is also the time frame when rapid adipose tissue development occurs postnatally (53). The heart of fatVHLko mice showed enlarged atria, ventricles, and wall thicknesses. Cardiomyocyte hypertrophy and increased numbers of Ki67^+^ cells, along with increased cardiac angiogenesis, were apparent in the heart. There was also fibrosis in the heart of fatVHLko mice. Functions of the fatVHLko heart were also compromised with reduced ejection fraction, increased end diastolic pressure, and decreased dynamic regulation of blood pressure, especially −dP/dt. The fatVHLko mice died in early adulthood with a median survival time of 10 weeks, likely due to sudden heart failure (17). This phenotype occurred in the homozygous fatVHLko mice only as *Vhl* heterozygous mice were developmentally indistinguishable from wild-type littermates. In contrast to this study, the *Phd2^f/f^/aP2-Cre* mice appeared to have normal cardiac development (46), whereas the heart phenotype was not examined in the aP2-*Phd2*KO mice (47). The differences between *Vhl* knockout and *Phd2* knockout could be attributed, in part, to the potential compensation by other PHD isoforms and the different strains of the aP2-cre mice, as discussed above.

As primary effectors of the hypoxia-signaling pathway, both HIF-1α and HIF-2α proteins are stabilized with *Vhl* deletion, but they have non-overlapping functions (54). We further created mouse models with different combinations of adipocyte-specific double or triple knockout to determine the differential roles of HIF-1α and HIF-2α in the pathological progression of fatVHLko mice (17). Genetic deletion of both *Vhl* and *Hif2a* completely normalized the development of the fatVHLko mice with no apparent signs of any developmental or behavioral defects. In stark contrast, deletion of *Hif1a* together with *Vhl* (fatVHL1Ako) significantly exacerbated hypertrophic cardiomyopathy and resulted in much earlier onset of death with a median survival time of only 4 weeks for fatVHL1Ako mice (17). These results suggest that chronic activation of HIF-2α, but not HIF-1α, in adipocytes is the main cause of adipose tissue pathology and hypertrophic cardiomyopathy. Consistent with these findings, cardiac abnormalities were not found in transgenic mice with adipocyte-targeted expression of the constitutively active HIF-1α-ΔODD (27) although the study by Lee et al. suggested otherwise (6).

The basal plasma glucose levels of fatVHLko mice were lower than those of their wild-type littermates. The fatVHLko mice showed by and large normal responses to intraperitoneal injection of glucose or insulin and showed moderate abnormalities in lipid metabolism. These findings suggest that the development of hypertrophic heart disease in pathologically obese subjects is likely independent of insulin resistance and/or hyperlipidemia, two commonly regarded key risk factors for cardiovascular diseases. Nevertheless, adipocyte-targeted deletion of *Vhl* led to both systemic and local adipose tissue inflammation. Expression of immunoglobulin genes, chemokines (*Ccl2*, *Ccl3*, *Ccl7*, and *Ccl8*), and cytokines (*Il1b*, *Il6*, and *Tnf*) was robustly increased in *Vhl*-deleted adipose tissue. Serum levels of IL-1β, IL-6, MCP-1/CCL2, and TNFα among others were also significantly increased in fatVHLko mice. Consistent with the systemic inflammatory phenotype, the NFκB pathway is strongly activated in the heart of fatVHLko mice (17). Furthermore, the NFAT pathway, a key player in development of cardiac hypertrophy (55), was also activated in the heart of fatVHLko mice (17). Consistent with these observations, it has been shown that NFκB can activate NFAT via protein–protein interaction to induce cardiac hypertrophy (56). Collectively, these findings strongly support the emerging concept that chronic inflammation is a leading cause of heart diseases (57–61).

Mechanistically, the inflammation phenotype was mainly dependent on activation of HIF-2α because double knockout of *Vhl* with *Hif2a*, but not with *Hif1a*, normalized expression of pro-inflammatory genes in adipose tissue and reduced activation of NFκB and the NFAT pathways (17). These results clearly demonstrate that HIF-1α and HIF-2α play fundamentally different roles in adipocytes. Importantly, chronic activation of HIF-2α in adipocytes elicits local inflammation in adipose tissue and increases production of circulating pro-inflammatory cytokines and chemokines, which then induces hypertrophic cardiomyopathy by activating NFκB and NFAT pathways.

Several similarities can be drawn between the fatVHLko models and clinical obesity with regard to the heart phenotype. Both clinical and epidemiological studies have shown that obesity is associated with left ventricle dysfunction and heart failure independent of diabetes, hypertension, and coronary artery disease (52). Some obese patients develop ventricular hypertrophy and congestive heart failure without other comorbidities in the heart (51, 62) while others present with diastolic dysfunctions but without hypertension (63). Similarly, the fatVHLko and fatVHL1Ako mice develop lethal cardiomegaly without presenting with severely compromised heart functions except reduced diastolic −dP/dt or significant metabolic abnormalities including insulin resistance and hyperlipidemia (17). In light of adipose tissue hypoxia associated with severe obesity (7, 10, 27), our study has led to a new disease mechanism underlying the etiology of obesity-associated cardiomyopathy. As this model suggests, obesity-induced pathological changes adipose tissue, such as adipocyte hypertrophy, decreased O_2_ supply, and increased O_2_ consumption, lead to the development of adipose tissue hypoxia that, in turn, induces sustained activation of HIF-2α in adipocytes, which then results in both local and systemic inflammation, and contributes to the development of hypertrophic cardiomyopathy.

## Summary

The various genetic mouse model studies discussed above have illustrated an important role of the HIF pathway in adipose tissue under both physiological and pathological conditions, despite a lack of consistent observations in some of the studies. New genetic approaches would be required to further clarify these inconsistent results. Nonetheless, the current studies have presented strong evidence showing that HIF pathway can play a critical role in the development of adipose tissue inflammation and other pathological abnormalities reminiscent of obesity-induced diseases including obesity-associated hypertrophic cardiomyopathy. Pharmacological intervention of the HIF pathway will likely have the potential to control the progression of obesity and obesity-related symptoms.

## Conflict of Interest Statement

Qun Lin and Zhong Yun declare that the research was conducted in the absence of any commercial or financial relationships that could be construed as a potential conflict of interest.
